# Anticancer effect of physical activity is mediated by modulation of extracellular microRNA in blood

**DOI:** 10.18632/oncotarget.27609

**Published:** 2020-06-02

**Authors:** Alessandra Pulliero, Ming You, Pradeep Chaluvally-Raghavan, Barbara Marengo, Cinzia Domenicotti, Barbara Banelli, Paolo Degan, Luigi Molfetta, Fabio Gianiorio, Alberto Izzotti

**Affiliations:** ^1^ Department of Health Sciences, University of Genoa, Genoa, Italy; ^2^ Center for Disease Prevention Research, Medical College of Wisconsin, Milwaukee, WI, US; ^3^ Department of Obstetrics and Gynecology, Medical College of Wisconsin, Milwaukee, WI, US; ^4^ Department of Experimental Medicine, University of Genoa, Genoa, Italy; ^5^ IRCCS Ospedale Policlinico San Martino, Genoa, Italy; ^6^ Orthopaedics-Rehabilitation Unit, Department of Neuroscience, Rehabilitation, Ophthalmology, Genetics and Maternal-Infantile Sciences, University of Genoa, Genoa, Italy; ^7^ Department of Internal Medicine, University of Genoa, Genoa, Italy

**Keywords:** circulating microRNAs, sports and exercise, carcinogenesis, cancer prevention, microRNA transfection

## Abstract

Epidemiological studies provide evidence that physical activity reduces the risk of cancer, particularly of breast cancer. However, little is known about the underlying molecular mechanisms as related to microRNAs. The goal of the herein presented study is to explore the involvement of miRNAs in beneficial effects exerted by physical activity in breast cancer prevention. Thirty subjects (mean age: 57.1 ± 14.7 years) underwent 45 minutes of treadmill walking under standardized conditions. The levels of extracellular miRNAs were evaluated in blood plasma before and after structured exercise by means of microarray analysis of 1,900 miRNAs identifying mostly modulated miRNAs. Structured exercise has been found to modulate the expression of 14 miRNAs involved in pathways relevant to cancer. The different expression of two miRNAs involved in breast cancer progression, i. e. up-regulation of miR-206 and down-regulation of anti-miR-30c, were the most striking effects induced by exercise. The biological effects of these miRNAs were investigated in MCF-7 human breast cancer cells. miR-206 transfection and anti-miR-30c silencing, inhibited cell growth and increased apoptosis of MCF-7 cells. Moreover, the combined use of the two miRNAs further enhanced apoptosis and induced growth arrest in the G1/S phase of cell cycle. Our results support that physical activity effectively change the expression of extracellular miRNAs. Specifically, miR-206 up-regulation and anti-miR-30c down-regulation act as suppressors in breast cancer cells. The evaluation of these miRNAs in blood can be used as non-invasive biomarkers for breast cancer prevention.

## INTRODUCTION

The relevance of structured exercise for public health has been addressed by the World Health Organization, and its lack is estimated to be the main risk factor for 21–25% of breast and colon cancer cases, 27% of diabetes cases, and 30% of ischemic heart disease cases [1, 2]. Several epidemiological studies demonstrated the dose-dependent protective effect of regular and moderate structured exercise against chronic degenerative diseases, with a particular reference to both the primary (prevention of cancer onset) and tertiary (prevention of cancer relapses) prevention of cancer [2]. These findings refer only to structured physical activities, i. e. those proposed according to international guidelines such as those proposed by the American College of Sports and Medicine [3]. Breast cancer survivors engaging in structured exercise increase the drainage of lymph from their upper limbs, thereby decreasing the side effects of mastectomy, significantly lowering their risk of a cancer relapse and improving their immune functions [4]. Conversely, no beneficial effects of an unstructured structured exercise (e. g., household work) have been observed [5]. Moderate-intensity aerobic exercise combined with resistance training benefited early adjuvant breast cancer treatment; after 18 weeks of an exercise program, there was a positive effect on physical fatigue, cardiopulmonary function, and muscle strength among participating patients [6].

Randomized clinical trials have shown that structured exercise interventions can change biomarkers of cancer risk [7].

The main preventive molecular mechanisms activated by structured exercise, include regulation of insulin-like growth factors (IGFs) and aromatase inhibition. Insulin resistance, hyperinsulinaemia, hyperglycaemia and type 2 diabetes have been linked to increased risk of breast, colon, pancreas and endometrial cancers. Structured exercise improves insulin resistance, reduces hyperinsulinaemia and reduces risk for diabetes, which could explain the link between increased structured exercise and reduced risk for these cancers [8].

A lack of structured exercise enhances the activity of aromatase, an enzyme mainly locates in white adipose tissue responsible for a key step in the biosynthesis of estrogens, thus increasing estradiol levels in the serum, a major risk factor for breast cancer onset and progression. Recent findings indicate that women with a history of breast cancer who engage in more than 9 metabolic equivalent (MET)·h/week of structured structured exercise (corresponding to 3 h per week of brisk walking) after breast cancer diagnosis had a significantly lower risk of death or breast cancer recurrence than women who were physically inactive [9, 10].

However, the mechanisms underlying the preventive effect of structured exercise against cancer have been only partially depicted. MicroRNAs (miRNAs) play pivotal roles in carcinogenesis and cancer outcomes [11], including breast cancer [12]. miRNAs function as regulators of myogenesis and muscle mass, and structured exercise modulates miRNA expression, especially in skeletal muscle. These muscle-specific miRNAs are known as ‘myomiRs’ [13]; among them, miR-1, miR-133a, miR-133b, and miR-206, account for nearly 25% of miRNA expression in skeletal muscle in both humans and mice [13]. Structured exercise adaptively changes the level of circulating miRNAs in animals and human beings [13] and this effect is related to the different type of structured exercise [14].

Resistance exercise is associated with the down-regulation of miR-1 and miR-133a, leading to increased IGF-1/AKT signaling. Endurance exercise leads to different adaptive responses which are regulated by miRNAs, including mitochondrial biogenesis, angiogenesis and fiber-type shifts to an oxidative metabolic profile [14]. Incubation of MCF-7 estrogen responsive breast cancer cells and MDA-MB-231 triple negative breast cancer cells treated with post-exercise serum, from both healthy volunteers [15] and operated cancer patients [16] resulted in a reduction of breast cancer cell viability in comparison with breast cancer cells incubated with pre-exercise sera. MCF-7 and MDA-MB-231 challenge with post-exercise sera from operated cancer patients receiving adjuvant chemotherapy compared to pre-exercise serum leads to a viability reduction of 11% in MCF-7 cells and 9% in MDA-MB-231 cells [15]. Furthermore, the viability of both breast cancer cell lines supplemented with sera from healthy women was also significantly reduced by the exercise-conditioned sera, resulting in 19% reduction in MCF-7 viability and 13% reduction in MDA-MB-231 viability by 2 h treatment with post-exercise sera.

Pre-incubation for 48 hours with post-exercise sera from healthy volunteer’s decrease cancer incidence and growth in nude mice inoculated with MCF-7 or MDAMB-231 breast cancer cells. In particular, cancer incidence decreased from 90% to 45% and cancer volume was reduced by 76% [15].

Based on these premises, we set up the herein reported study aimed at identifying the contribution of extracellular miRNAs to the anti-cancer properties of blood serum conditioned by structured exercise in humans. Accordingly, we analyzed circulating miRNAs expression profiles before and after structured exercise and evaluated their potential anti-cancer properties in breast cancer cells. The aim of the study was to develop new, non-invasive biomarkers that could provide information on the beneficial effects of structured exercise in cancer prevention.

## RESULTS

### Subject characteristics: blood glucose, heart rate, blood pressure and metabolic equivalent

**Figure 1:**
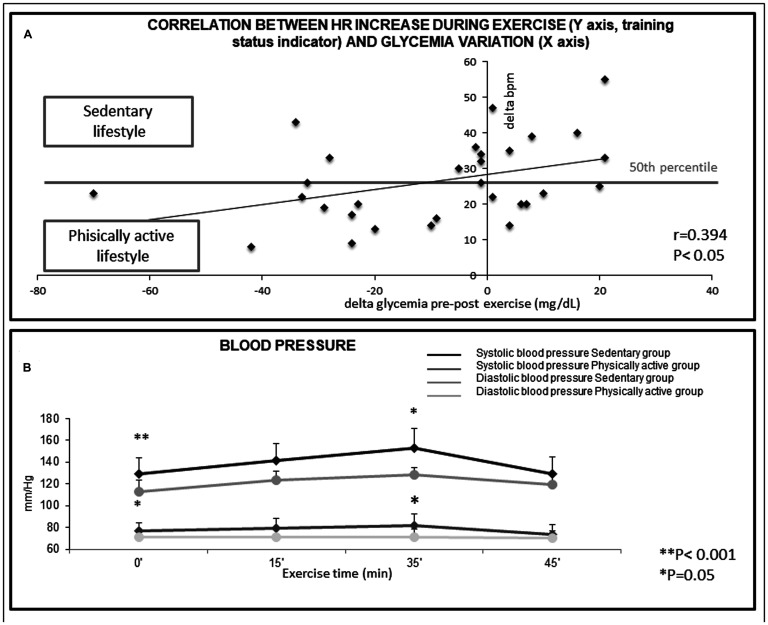
(**A**) Correlation between Heart Rate increase during exercise (Y axis, training status indicator) and glycemia variation (X axis) in the 30 subjects. The *Beats Per Minute* measurement (bpm), indicates that exceedingly high heart-rate increase during physical activity in non-trained subjects are not useful to decrease glycemia. R = 0.394, *P* < 0.05. (**B**) Variations in blood pressure during structured exercise. Reported values were relative to the start of the exercise (basal, 0 minutes), after 15 minutes, at the period of maximum intensity (peak, 35 minutes) and at the end of the exercise (45 minutes). ^**^ *p <* 0.001, ^*^ *p <* 0.05, as compared to controls.

Training status of each subject was evaluated by questionnaire: subject performing at least 30 min 3 days per week of physical activity (i. e., brisk walking, gym, bike) were classified as physically active; otherwise as sedentary. 18 subjects were physically active and 12 sedentary. 3 subjects despite being classified as physically active by questionnaire underwent heart rate increase >25 bpm (pulse per minute) thus being located in the horizontal central line in Figure 1.

The influence of training status was also determined by evaluating the difference in heart beats per minute (bpm) between pre and post exercise. The relationship between the difference in heart beats per minute (bpm) between pre and post exercise on the alterations of blood glucose (delta glycaemia pre-post exercise) was examined by regression analysis (Figure 1A). As indicated by the regression line, in trained, physically active subjects, blood glucose was more markedly decreased than in untrained subjects that had a sedentary lifestyle (*P <* 0.05), in whom the glucose decrease after physical activity was almost undetectable (Figure 1A). The regression line indicates that in trained physically active subjects (i. e. those having delta bpm below the 50th percentile) blood glucose was more markedly decreased than in untrained subjects having a sedentary lifestyle (*P <* 0.05), (Figure 1A). This finding indicates that training status is important in modulating the efficacy of structured exercise to induce a reduction of blood glucose levels.

A statistically significant inverse correlation (*P <* 0.05) was found between the decrease in blood glucose and the increase in heart rate during exercise (Δ0’/35’), that is an indicator of the training status of the subjects. Indeed, trained subjects display smaller increases in heart rate during exercise and a faster recovery of their resting heart rate after stopping the exercise (Figure 1A).

Untrained (sedentary lifestyle) subjects showed significantly higher values for both basal and peak systolic blood pressure than trained (physically active lifestyle) subjects (*P <* 0.001 and *P =* 0.05, respectively). Similarly, basal and peak diastolic blood pressure values were higher in untrained subjects in comparison with trained subjects (*P =* 0.05 and *P =* 0.01, respectively) (Figure 1B).

Physical effort assessed by the Borg scale was different comparing untrained and trained subjects. The scores (mean ± SD) were 5.05 ± 0.25 for untrained subjects versus 2.63 ± 0.62 for trained subjects (*P <* 0.001). This finding shows that untrained subjects are less resistant to moderate structured exercise than trained.

In the untrained subjects having high basal blood pressure, structured exercise caused a greater increase in heart rate in respect to the other subjects. The increase in heart rate at the exertion peak (HR Δ0’/35’) was 35.22 ± 4.00 bpm in high-blood pressure subjects versus 21.81 ± 2.62 bpm in normal-BP subjects (*P <* 0.01).

A relationship was observed between blood pressure and a decrease in blood glucose after structured exercise. High-blood pressure subjects displayed a change in blood glucose pre-post exercise of +0.89 ± 5.81 mg/dl, whereas normal-blood pressure subjects had a change of –20.91 ± 7.55 mg/dl (*P <* 0.05). These data indicate that the beneficial effects of structured exercise should be determined taking into account the specific characteristics of the subject.

Physically active lifestyle influenced the variation of systolic arterial blood pressure during the exercise. Indeed, a statistically significant increase (Δ0-35 min systolic blood pressure, *P =* 0.05) was found comparing sedentary and trained subjects (Figure 1B).

### Plasma miRNA and structured exercise

**Figure 2:**
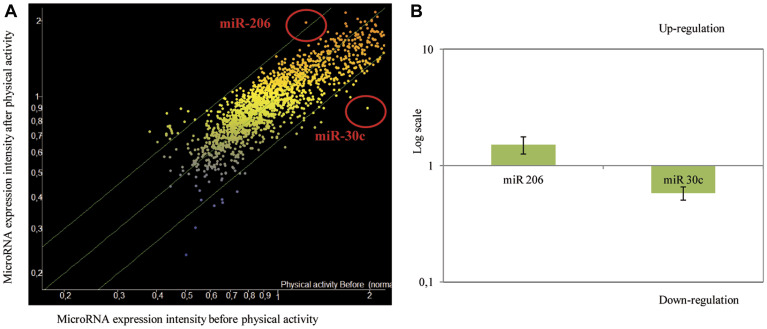
(**A**) Scatter plot reporting variations of miRNA expression before and after physical activity as evaluated by microarray in 30 subjects testing 1,900 microRNAs in blood plasma. MiRNAs, whose expression was modified by physical activity, were identified as red dots in scatter-plot. (**B**) Up-modulation of miR-206 and down-modulation of miR-30c in transfected MCF7 cells (logarithmic scale). qPCR analysis of miR-206 and miR-30c expression after transfection of miR-206 mimics and miR-30c siRNA. The data were reported as variation in respect to their expression in the control. The fold change in the samples (MCF7 cells transfected with miRNA-mimic and/or siRNA) was normalized to the reference RNU6 and expressed relative to a calibrator sample (mock MCF7 sample) using the 2−(ΔΔCt ± SD) method.

The expression of miRNAs in the plasma collected from subjects before and after structured exercise was measured using microarray analysis. Structured exercise modified the expression profile of extracellular miRNAs (Figure 2A). Fourteen different miRNAs showed variations of their expression before and after structured exercise based on the previously stated criteria (volcano plot, k-nearest neighbor algorithm). The specific miRNAs and the associated biological functions, as inferred from the available literature, are reported in Table 1. The miRNAs, involved in the prevention of cancer were all down-regulated by structured exercise with the exception of miR-206, which was up-regulated. The miRNAs mostly modulated by structured exercise were miR-206 (up-regulated) and anti-miR-30c (down-regulated).

**Table 1:** Parameters of the exercise protocol performed: metabolic equivalent (MET) blood pressure, heart rate

	Minutes (min)	Speed (km/h)	Incline (%)	MET (ml O2/kg/min)	Blood pressure (mm/Hg)		Heart rate (bpm)
					Systolic	Diastolic	
**Warm-up**	0	2	0	1.95	125.2 ± 15.1	75.6 ± 7.7	73.5 ± 9.1
	3	2	1	2.12			
	5	2.7	1	2.53			
**Central part**	10	2.7	2	2.76			
	15	3.2	2	3.08	136.8 ± 16	77.3 ± 9.1	85.3 ± 9.4
	20	3.2	3	3.36	146.3 ± 19.3	78.8 ± 10.8	99.8 ± 11.8
	25	3.5	4	3.86			
	30	3.5	5	4.16			
	35	3.8	5	4.43			
**Cool-down**	40	3.2	3	3.36			
	45	2.7	0	2.29			
**Recovery**	46	0	0	0	126.8 ± 14.7	73.0 ± 7.8	81.8 ± 11.9
**MET arithmetic mean**				2.83			
Δ **between physical activity’s start-time and effort peak (0’/35’)**	Δ 0/35				20.7 ± 14.1	3.2 ± 6.5	26.5 ± 11.4
Δ **between effort peak and recovery-time (35’/46’)**	Δ 35/46				−19.8 ± 12.3	−5.8 ± 8.3	−18.6 ± 10.7

Data were measured before the activity session (0 minutes), after 15 and 35 minutes, at the beginning of the recovery period (35-45 min), and at the end of the treadmill workout (46 minutes). The metabolic equivalent (MET) was calculated indirectly by means of an equation based on speed and incline of the treadmill (id est; [(mph ×26.8) x (0.1 + ((Grade × 0.018)) + 3.5]/3.5).

The relationships of the miRNAs modulated by structured exercise with the measured clinical variables are reported in Table 2. A strong relationship was found with the decrease in glucose: 9 out of the 15 miRNAs were modulated only in patients showing a decrease in glucose as a result of structured exercise. This finding indicates that the down-regulation of these miRNAs reflects the beneficial effects of structured exercise in terms of reducing blood glucose, which primarily was found in subjects that were already trained. Similarly, 9 miRNAs were related to increase in the diastolic pressure at 35 minutes and to the level of exertion as evaluated by the Borg scale. These findings indicate that the benefits of miRNAs are affected by the training status of the subject, as it is also indicated by the relationship of the modulated miRNAs with a low basal heart rate (Table 2).

**Table 2:** Characteristics and biological functions of the extracellular serum miRNAs modulated by physical activity

miRNA	Fold variation	*P* value	Biological function	Tissue specificity	Drug modulation	References
miRNA-25	2.65 ↓	0.0210	Dicer regulation; Oncogene (PTEN) suppression; calcium accumulation in mitochondria; apoptosis; mitochondrial function; suppression of colon cancer cells proliferation; oncogene (SMAD 7/ TGF β) suppression	—		[41]
miRNA-30c	2.25 ↓	0.0235	Protein repair; cell proliferation; stem cell recruitment; multi-drug resistant; stress (NFkB) response; oncogene (BCL9) suppression	Lung	Modulated by Metformin in mouse lung	[33, 34]
				Breast		
				Ovary		
miRNA-92b	2.16 ↓	0.0495	Dicer regulation; cell proliferation; apoptosis; Oncogene (PTEN) suppression	Lung		[43]
miRNA-133b	2.01 ↓	0.0269	Muscle response to physical activity; associated with heart failure; oncogene (EGF) suppression; cell proliferation; cancer invasion	Skeletal muscle	
				Heart	
miRNA-204	4.08 ↓	0.0170	Epithelial mesenchymal transition; apoptosis; cell proliferation; oncogene (VHL) suppression; removal of cancer cells by mitochondrial driver autophagy; regulation of autophagy induced by ischemia-reperfusion in heart (rat)	—		[40, 44]
miRNA-206	2.21 ­↑	0.0297	Muscle response to physical activity; associated with breast cancer prognosis and patients survival; oncogene (Wnt) suppression; cell proliferation; cardiac regeneration; inhibition of cancer invasion; metalloprotease (TIMP-3) inhibition	Skeletal muscle		[33, 36]
				Breast		
miRNA-450a	2.22 ↓	0.0048	—	Heart	Decreased in heart of diabetic mice	[8]
miRNA-492	2.56 ↓	0.0110	Angiogenesis	Blood vessels (endothelium)		[46]
miRNA-513b	2.11 ↓	0.0378	Inflammation; apoptosis	Lymphoma		
miRNA-516b	2.56 ↓	0.0053	Metastasis suppressor	Melanoma		[12]
miRNA-519e	2.22 ↓	0.0078	Cell proliferation; Oncogene (HuR) suppression	—		[45]
miRNA-711	3.14 ↓	0.0277	Inhibition of heart fibrosis after heart infarction	—	Modulated by Pioglitazone in rat heart	[39]
miRNA-765	2.13 ↓	0.0396	Oncogenic protein (HMGA1) suppression	Prostate		[47]
miRNA-877	2.06 ↓	0.0163	—	Hepatocellular carcinoma	Pioglitazone (2,10 up regulation in mouse lung)	

### miR-206 and miR-30 modulate viability and apoptosis in MCF-7 cancer cells

In order to evaluate the biological effects of anti-miR-30c downregulation and miR-206 upregulation on cancer cell growth, we silenced and over-expressed these two miRNA, respectively, in MCF-7 human breast cancer cells. In detail, the expression of miR-206 was increased by 1.51-fold while anti-miR-30c expression was reduced by 1.72-fold in respect to mock control (Figure 2B).

**Figure 3:**
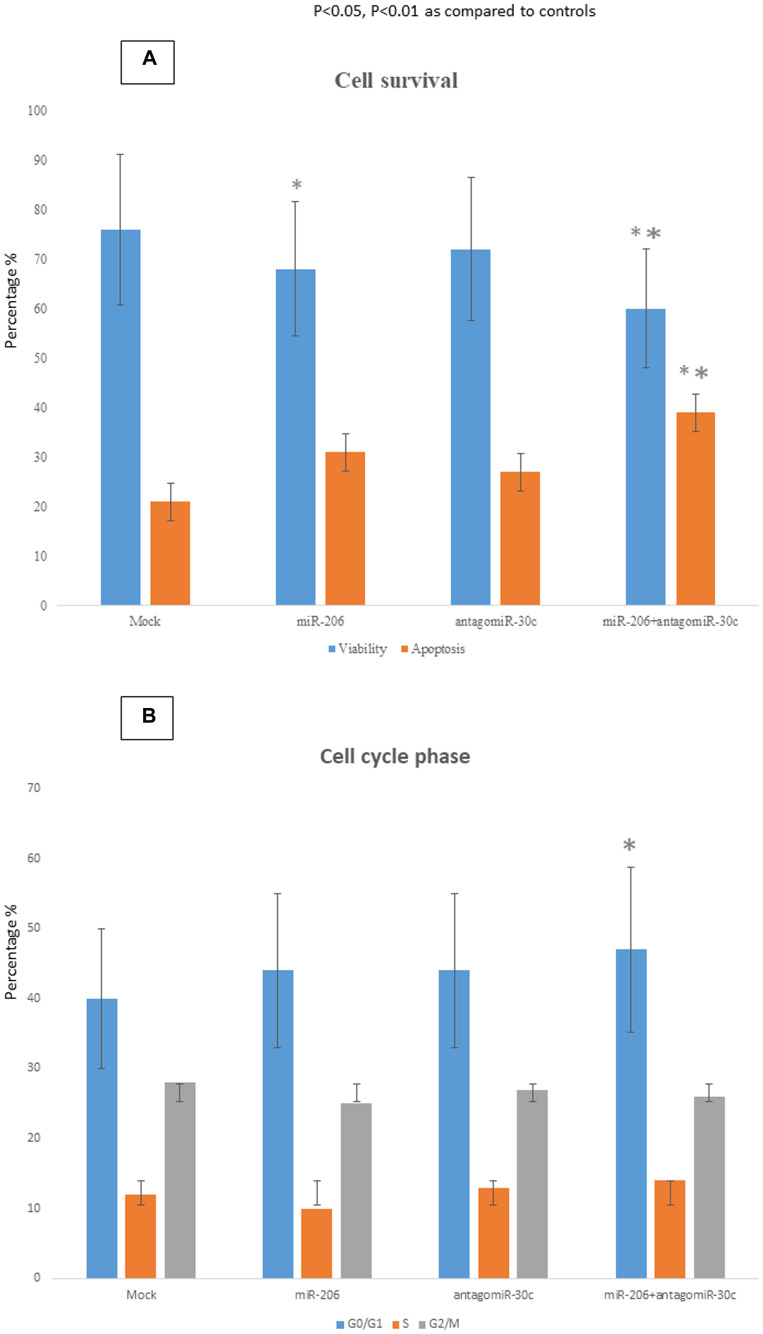
Biological effects of miR-30c and miR-206 modulation in breast cancer cells. Apoptosis (**A**) and cell cycle distribution (**B**) were analyzed in MCF-7 cells over-expressing miR-206 or silenced for miR-30c. The apoptotic rate and cell cycle phases were evaluated by cytofluorimetric analyses performed 48 h after the transfection. The data were reported as percentage. ^*^ *p <* 0.05 vs mock cells; ^**^ *p <* 0.01 vs mock cells.

In MCF-7 cells transfected with miR-206 a 16% reduction of cell viability and a concomitant 47% increase of apoptosis was observed in respect to mock cells (Figure 3A). Anti-MiR-30c silencing alone barely affected either viability or apoptosis. However, when used in combination, miR-206 transfection and anti-miR-30c silencing significantly decreased viability and increased apoptosis of MCF-7 cells (26% reduction of cell viability; 80% increase of apoptosis in respect to mock cells) (Figure 3A). Use of miR-206 or anti-miR-30c alone did not cause any significant alteration of the percentage of cells in GO/G1, S or G2/M phases (Figure 3B). Conversely, the simultaneous use of both miR-206 and anti-miR-30c led to an increase of 15% in the number of cells blocked in G0/G1 phase.

### Myoblast and breast cancer cells

**Figure 4:**
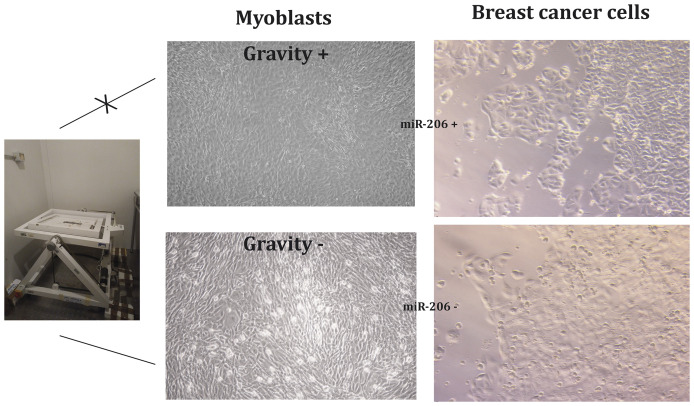
Myoblasts stimulated by gravity (G) release miRNA-microvesicles inhibiting breast cancer cell growth.

Figure 4 reports the results obtained comparing the effects of supernatant extracted from myoblasts either un-activated or activated by microgravity on MCF7 breast cancer cells.

The presence of miR-206 in the supernatant extracted from activated myoblasts was confirmed by qPCR (data not shown). Microscope analysis demonstrate: (a) the different morphology of activated and un-activated myoblasts; (b) the different behavior of cancer cells; indeed, these cells when exposed to supernatant from activated myoblasts undergo slowing down of cell growth (no confluence) and remarkable increase of apoptosis as highlighted by light-reflecting droplets corresponding to death cells detached from the basement. MTT viability test preformed on MCF7 cells highlighted a 38% decrease of viable cells when treated with supernatant collected from gravity-activated myoblasts.

## DISCUSSION AND CONCLUSIONS

Structured exercise has a beneficial role in many diseases and in particular it has been shown to contribute to reduce the onset and to improve the prognosis of cancer [17–19]. This effect is related to the fact that structured exercise not only acts on skeletal muscle but also at a systematic level and, consequently, it can also influence tumor microenvironment and cancer hallmarks [20]. Among the most evident systemic effects found in the subjects analyzed in our study, a reduction of blood glucose levels has been reported after structured exercise. This result is in line with a recent work demonstrating that structured exercise induces a decrease in the hematic levels of glucose, insulin and IGF-1, by increasing the muscle glucose uptake [21].

In our study, structured exercise decreases blood glucose primarily in trained subjects while structured exercise in non-trained subjects is not effective in decreasing blood glucose level. These finding is likely related to the expression of GLUT4 glucose receptor that is established to occur mainly in physically activated skeletal muscle [22].

The ability of structured exercise to modulate miRNAs relevant to cancer prevention has not been well investigated. Although some authors have hypothesized that structured exercise could modulate miRNAs with cardiovascular and onco-protective relevance [23], any direct evidence in human beings has been reported until now. Therefore, this study aims to explore this issue.

Firstly, our results indicate an overall trend of miRNA down-regulation, with 13 out of the 14 miRNAs that were significantly down-regulated by structured exercise. Since miRNA overload is linked to the activation of inflammatory pathways and lymphocyte activation through TLR3/7 binding [24], structured exercise by modulating miRNA in the blood might exert an anti-inflammatory effect. miRNAs are silencers of gene expression by coupling with complementary messenger RNA and consequent RISC activation. Whenever gene expression has to be unlocked, miRNAs are typically mainly downregulated. Because of this reason, downregulation prevails in case of environmental chemical stress [25, 26].

Indeed, physical activity induces a short-term oxidative stress rebounding after few hours in the activation of anti-oxidant pathways [27].

We have found that structured exercise modulates the expression of miR-206 and miR-133, two muscle miRNAs whose levels are related to IGF and IGF-1R expression [28]. Furthermore, the results of our functional analyses provide evidence that miR-206 inhibits breast cancer cells growth by blocking the G1/S transition and suppress cell proliferation.

MiR-206 expression was correlated with negative ER status, negative PR status, and negative HER-2 status (*P <* 0.05), regardless of age, menstruation, lymph node metastasis. miR-206 has been demonstrated to be decreased in normal breast tissues compared with primary breast tumors and in ER-positive compared with ER-negative tumors [29]. However, our females were all at postmenopausal age; accordingly, the influence of estrogenic status on the results obtained is expected to be negligible.

Physical activity unlocks the expression of many genes in skeletal muscle, mainly including those encoding for enzymes involved in oxidative phosphorylation, mitochondrial functions and antioxidants activities. Indeed, as demonstrated by biopsy, physical activity results in a dramatic upregulation of genes associated with mitochondrial function in skeletal muscle [30]. Our results indicate that these genes unlock is triggered by an overall trend towards miRNA downregulation, as detected in blood plasma.

The effects of physical activity on miRNAs bearing role in cancer prevention depend on their specific activity. miRNAs directly silencing oncogenes are upregulated, as demonstrated for miR-206 an established wnt oncogene silencer. Conversely, other miRNAs acting as negative regulator of genes blocking biological processes involved in cancer development are downregulated. This downregulation corresponds to the unlocking of controlled genes that can accordingly express their defensive activities. This situation was envisaged by our results for various biological function including NFkB inflammation silencing, oncogene suppression by activation of anti-oncogenes, decrease of cell proliferation. Structured exercise is able to modulate the expression of miRNAs that have been shown to be protective against cancer. We have demonstrated this modulation exert after 45 minutes of structured exercise. MiR-206 is down-regulated in human breast cancer [31] and in particular in those resistant to tamoxifen, and the down regulation is associated with advanced clinical stage of breast cancer [32], In the present study we found that miR-206 is up-regulated by structured exercise. The enhanced expression of miR-30c is associated with ER-positive breast cancers [33]. Our findings, according to literature, show that miR-30c is down-regulated by structured exercise, thus supporting the relevance of structured exercise in the tertiary prevention of breast cancer. Indeed, anti-miR-30c promotes the invasive osteoclastic phenotype of metastatic breast cancer, explaining the clinical observation of poor survival of patients with elevated miR-30c levels [34].

Our *in vitro* results have been obtained using a ER+ breast cancer cell line (MCF7. *In vitro* experiments evaluating the effect of physical activity on different breast cancer cell lines have been performed by other authors using exercise-conditioned human blood serum [35]. These authors demonstrated that exercise-conditioned sera significantly reduced the ability of triple negative breast cancer cells (MDA-MB-231) to form colonies in soft agar, in comparison to pre-exercise sera collected from the same subjects.

Our results provide evidence that miR-206 together with anti-miR-30c, cooperatively act to reduce cancer cell growth by inducing apoptosis and slowing G1/S transition. This result is in line with recent studies demonstrating a close relationship between miR-206 and cyclin D2, a G1/S specific cyclin, whose activity is required for cell cycle G1/S transition, reflecting a pivotal role of this muscle-specific miRNA in cancer prevention [32, 36]. Different studies have described an inverse relationship between miR-206 and cyclin D2 expression, confirming its possible role as a predictor of breast cancer [37, 38]. The miRNAs that we have observed to be modulated by structured exercise are involved in cancer prevention mechanisms including oncogene suppression (miR-25, miR-30 miR-92, miR-133, miR-204, miR-206, miR-519), reduction of inflammation (miR-513), inhibition of cell proliferation (miR-25, miR-92, miR-133, miR-204, miR-206, miR-519), induction of apoptosis (miR-25, miR-204, miR-513), inhibition of stem cell recruitment (miR-204), multidrug resistance (miR-30), invasion and metastasis (miR-133, miR-206) [39–44].

Myoblasts stimulated by gravity (G) release miRNA-microvesicles containing miR-206 thus inhibiting breast cancer cell growth. Lack of gravity stimulation corresponding to the lack of physical activity, de-differentiate myoblast transforming them into rounded the cells undergoing decreased expression of myosin related proteins and inhibit miRNA release as well as the ability of myoblast to inhibit breast cancer cell proliferation. This finding provide evidence of the direct origin of miR-206 and anti-miR-30 from myoblast as well as their preventive effect towards breast cancer cell growth [45].

In conclusion, this study provides evidence that miRNA modulation is a specific molecular mechanism through which structured exercise exerts preventive effects against cancer. The possibility of using these two miRNAs for breast cancer prevention is of interest. MicroRNA as delivered by lipid nanoparticles has been already be effective in mice in preventing NNK induced lung cancer [46]. However, insofar no similar experiments exist as far as concern breast cancer prevention.

Moreover, the evaluation of miR-206 and anti-miR-30c levels in the blood of breast cancer patients could be useful as non-invasive biomarkers in guiding future strategies for cancer prevention.

## MATERIALS AND METHODS

### Subjects

Thirty female subjects were recruited into the present study. All subjects were Caucasians, healthy, resided in northwestern Italy, and were between 54 and 78 years old, i. e. at post-menopausal age. Subjects were categorized according to training status. Inclusion criteria were: healthy subjects, no drug assumption; exclusion criteria: adverse health conditions, any diagnosis of systemic diseases. The influence of these variables on the following end points was tested: heart rate, blood pressure, blood glucose concentration, and extracellular miRNAs present in the blood plasma. Peripheral venous blood (6 ml) was collected from each subject into vacutainers both before and immediately after performing the structured exercise test. All participants gave their informed, written and signed consent. All information regarding participants was rendered anonymous after the collection of personal data and blood samples. The study was approved by the Ethics Committee of the San Martino Hospital on October 12th 2010.

### Structured exercise training program

The exercise protocol consisted of walking at a moderate intensity on a treadmill. The treadmill exercise session included a 5-minute warm-up, regular exercise training for 45 minutes, and a 10-minute cool down phase. The training protocol was designed to simulate brisk walking, which is often suggested as the first step to beginning an active lifestyle to reduce the risk of chronic health conditions [16]. During the training program, subjects were monitored for blood pressure and heart rate, which were measured at 0, 15, and 35 minutes and at the end of the activity. Blood glucose was tested before and immediately after structured exercise. The exercise test protocol was structured according to the parameters: metabolic equivalent (MET) blood pressure, heart rate. Data were measured before the activity session (0 minutes), after 15 and 35 minutes, at the beginning of the recovery period (35-45 min), and at the end of the treadmill workout (46 minutes). The metabolic equivalent (MET) was also calculated for each change of speed and/or grade of the treadmill during protocol exercise. MET was calculated by an equation based on speed and incline of the treadmill: [(mph ×26.8) x (0.1 + ((Grade × 0.018)) + 3.5]/3.5). The structured exercise protocol was structured according to an incremental model complemented by a cool-down phase. The mean value of metabolic equivalent (MET) of the exercise proposed corresponds to 2.8 MET (Supplementary Table 1), in accordance with the MET score group values for the range of low-impact exercises (0-3 MET) [47]. The physical condition of the subjects was monitored during the test. At 35 minutes (the point of maximum functional effort), they were asked to quantify their level of fatigue according to the Borg scale (from 1 to 10). Treadmill exercise was preferred to cycle ergometers because in this last exercise fragile subjects often interrupt prematurely due to muscle strain and fatigue. Furthermore, the use of a treadmill allows a greater accuracy than bike in the administration of the workloads by changing speed and inclination.

### Plasma miRNA analysis

Extra-cellular miRNAs were extracted using a commercially available kit (miRNeasy Mini Kit, Exiqon) from two aliquots of plasma obtained from each subject before and after the structured exercise session. The structural integrity of the miRNAs was evaluated via capillary electrophoresis (Agilent Bioanalyzer 2100). Total RNA was added to a reaction mixture in a final volume of 12 μl containing 1 μg of [3 ‘(N) 8-(A) 12-biotin-(A) 12-biotin 5′] oligonucleotide primer. The mixture was incubated for 10 minutes at 70° C and cooled on ice. Then, 4 μl of 5× first-strand buffer, 2 vol 0.1 M DTT, 1 μl of 10 mM dNTP mix, and 1 μl Superscript ™ II RNaseH - reverse transcriptase (200 U / μl) were added. The samples were incubated for 90 minutes at 37° C. After the incubation for first-strand cDNA synthesis, 3.5 μl of 0.5 M NaOH/50 mM EDTA was added to 20 μl of the first-strand reaction mixture containing fluorochrome Cy3/Cy5. The reaction mixture was incubated at 65° C for 15 minutes to denature RNA/DNA hybrids and to degrade RNA templates. Then, 5 μl of 1 M Tris-HCl (pH 7.6) was added to neutralize the mixture, and the samples were frozen at –80° C until use for chip hybridization.

The expression of miRNAs in the plasma was determined using commercially available microarrays (Exiqon), which contain 1,900 probes for human miRNAs. Microarrays were hybridized in 6× SSPE/30% formamide at 25° C for 18 h and washed in TNT (Exiqon) 0.75× at 37° C for 40 minutes. The microarrays were then analyzed to detect the emitted fluorescence using a laser scanner (Perkin Elmer Lite ScanArray® XL5K). The specifications of the scan were as follows: laser power: 80%, photomultiplier: 70%, resolution: 10 μm. The results were statistically analyzed using Genespring software (Agilent Technologies). The results for each miRNA analysis were determined based on averaging the values from two spots analyzed in duplicate after logarithmic transformation, subtraction of the background signal surrounding the spot, and the normalization of the data to the average of the results obtained for each microarray. Significant variations in miRNA expression before and after structured exercise were evaluated using a volcano plot analysis, with a 2-fold variation in expression as the threshold and *P <* 0.05 as the limit for significance. MiRNAs whose expression was modified by structured exercise were also identified using the k-nearest neighbor algorithm.

### Cell culture and miR-206 and siR-miR-30c transfection

MCF-7 (Cell Factory IRCCS San Martino IST Genoa Italy, ICLC code HTL95021) adherent cells were grown in DMEM (high glucose with L-glutamine) supplemented with 10% FBS (Gibco BRL Life Technology USA), 5% L-Glutamine (Gibco, BRL Life Technology USA), 1% penicillin (10,000 units), and 10 mg/mL streptomycin (Gibco, BRL Life Technology USA). Cells were grown at 37° C with 5% CO_2_ in a humidified incubator. MCF-7 (1.3 × 10^5^ cells per well) were seeded in a 6-well plate in 2 ml of an appropriate culture medium containing serum and antibiotics.

MiRNA mimic 206 and anti-miR-30c (Qiagen, Milano, Italy) were diluted to obtain a final concentration of 10 and 50 nM, respectively and transfected into the cells utilizing Lipofectamine 2000 (Invitrogen, Life Technology USA) according to the manufacturer protocol. Single and combined mimic/inhibitor transfections were performed. Cells were utilized for functional or expression analyses as described below, and the efficiency of the transfection was determined by flow cytometry 48 hr post-transfection, i. e. at the highest level of miRNA modulation as indicated by preliminary experiments. MCF-7 cells transfected with miR-206 and anti-miR-30c were harvested 48 h after transfection by trypsinisation. MiR-206 and anti-miR-30c expression was quantified by qRT-PCR analysis.

### Real time qPCR

Total RNA was retro-transcribed with miScript II RT kit (Qiagen) using miScript HiSpec Buffer. For each qRT-PCR reaction, were utilized 13 ng of retrotranscribed RNA. Amplification was performed in a volume of 10 μl containing miRNA-specific primers at 0.3 μM (miR-206 reverse: ACCTGCGTAGGTAGTTTCATGT, miR-206 forward: CGTCAGAAGGAATGATGCACAG; anti-miR-30c forward: 5′-GCCGCTGTAAACATCCTACACT-3′ and anti-miR-30c reverse: 5′-GTGCAGGGTCCGAGGT-3′) (Xia Y 2013) purchased from Tib MolBiol s. r. l. (Genova, Italy). RNU6 was utilized as an internal reference target (miScript PCR Controls Qiagen). Reactions were performed in triplicate using 2× Maxima SYBR Green qPCR Master Mix (Fermentas, St Leon-Rot, Germany).

qPCR reactions were performed under strictly controlled and standardized conditions using an epMotion 5070 liquid handling station (Eppendorf). A standard 3-step amplification with a 57° C annealing temperature was used. The fold change was normalized to the reference RNU6 and expressed relative to the calibration sample using the 2−(Δ Δ Ct ± SD) method. Specificity of the amplicon products was checked by analyzing melting curves.

### Evaluation of cell viability, cycle and apoptosis

These parameters were evaluated by cytofluorimeter analysis (Muse Cell Analyzer Millipore, USA) using the Muse Annexin V & Dead Cell Assay (EMD Millipore, USA). The analysis is based on the fact that the vital and non-vital cells are differently permeable to the two colorants present in the reagent and they are discriminated by the software “Muse ™ Count & Viability Software Module”.

Cell suspension (10^5^ and 10^7^ cells/ml) is diluted 1:20 with the reagent containing the two colorants and incubated in the dark at room temperature for 5 minutes. Cytofluorimeter analysis allows to evaluate the number of the nucleated and colored cells and to distinguish whole cells from cellular debris providing an accurate count of cell vitality percentages.

Apoptosis was evaluated by Annexin V & Dead Cell (Millipore) assay and using the Muse Cell Analyzer (Millipore) cytofluorimeter. The assay is based on the evaluation of morphological changes occurring during the apoptotic process. During the early stages of the apoptotic process, the phosphatidylserine (PS), which becomes outsourced from cell membrane, bind to Annexin V a protein with an high affinity for PS, specifically marking apoptotic cells. In combination with Annessin V, 7-AAD, a cell death marker that fails to color vital cells or early apoptosis cells is used as an integrity-indicator of cell membranes. Positivity to at least one, both or any of the two markers, distinguishes four possible cell types that are distinct in the plot resulting from MUSE analysis: non apoptotic cells (Annexin V-negative and 7-AAD negative); early apoptosis cells (Annexin V-positive and 7-AAD negative); late apoptotic cells and necrotic cells (Annexin V positive and 7-ADD positive); cellular and nuclear residues (Annexin V negative e 7-ADD positive). Cell suspension (10^4^ and 5^*^10^5^ cells / ml), was added to the same amount (100 μl) of the “Annexin V & Dead Cell” reagent. Each sample was incubated at room temperature for 20 minutes after and subsequently analysed by the cytofluorimeter using the specific apoptotic program.

The cell cycle was analyzed by using the Muse Cell Cycle Kit (Millipore). The assay is based on the use of propidium iodide (IP), a fluorescent DNA intercalated which is able to discriminate cells in relation to their DNA content: cells in Phase S have a doubled amount of DNA, so in G_2_/M phase the DNA content will be twice compared to “resting” cells (G_0_/G_1_). For each sample, 10^6^ cells of a suspension as monodispersed as possible, are centrifuged at 300 g for 5 minutes, re-suspended in PBS and centrifuged at 300 g for 5 minutes twice. The cell pellet is fixed and re-suspended in 1 ml of 70% ethanol. After at least 3 hours at –20° C the cells are centrifuged and re-suspended in PBS and centrifuged at 300 g for 5 minutes. The supernatant is removed and cells are re-suspended in 200 μl of Muse Cell Cycle Reagent containing IP. After 30 minutes of incubation at room temperature and in the absence of light, the sample is analyzed at the Mu se Cell Analyzer (Millipore).

### Myoblast and breast cancer cells co-culture

In order to verify that examined miRNA were effectively released for physically activated skeletal muscle an *in vitro* co-culture of myoblasts and breast cancer cells was set up. Myoblasts (CRL-1772; ATCC) were cultured for 24 h either activated by gravity (G+) or un-activated (G-) by exposing them to microgravity using a random position machine apparatus RPM (Dutch Space, Leiden, NL).

Supernatant was collected after 24 h and transferred to MCF7 cells whose morphology and behavior was evaluated by normal and light scattering microscopy.

### Statistical analysis

Data expressed as mean ± SE (standard error) were analyzed using SAS 8.2 software (Stat View., NC). Student’s *t* and ANOVA paired tests were performed to assess the statistical significance between groups. In all analyses, *p* ≤ 0.05 was considered to be statistical significance.

## SUPPLEMENTARY MATERIALS



## Abbreviations

IGFs: insulin growth factors
AMPK: 5′-adenosine monophosphate-activated protein kinase
mTOR: mammalian target of rapamycin
Ras/ERK: extracellular signal-regulated kinase
MyomiR: skeletal-muscle microRNA
AKT: protein kinase B

## References

[1] World Health Organization Global recommendation on structured exercise for health http://www.who.int/dietphysicalactivity/factsheet_recommendations/en 2010 Accessed 2010 (20 Avenue Appia, 1211 Geneva 27, Switzerland).

[2] Sanchis-Gomar F, Lucia A, Yvert T, Ruiz-Casado A, Pareja-Galeano H, Santos-Lozano A, Fiuza-Luces C, Garatachea N, Lippi G, Bouchard C, Berger NA Physical inactivity and low fitness deserve more attention to alter cancer risk and prognosis. Cancer Prev Res (Phila). 2015; 8:105–10. 10.1158/1940-6207.CAPR-14-0320. 25416409 PMC4315717

[3] Schmitz KH, Courneya KS, Matthews C, Demark-Wahnefried W, Galvão DA, Pinto BM, Irwin ML, Wolin KY, Segal RJ, Lucia A, Schneider CM, von Gruenigen VE, Schwartz AL, and American College of Sports Medicine American college of sports medicine roundtable on exercise guidelines for cancer survivors. Med Sci Sports Exerc. 2010; 42:1409–26. 10.1249/MSS.0b013e3181e0c112. 20559064

[4] Kim J, Choi WJ, Jeong SH The effects of physical activity on breast cancer survivors after diagnosis. J Cancer Prev. 2013; 18:193–200. 10.15430/jcp.2013.18.3.193. 25337546 PMC4189463

[5] Schnohr P, Grønbaek M, Petersen L, Hein HO, Sørensen TI Physical activity in leisure-time and risk of cancer: 14-year follow-up of 28,000 danish men and women. Scand J Public Health. 2005; 33:244–49. 10.1080/14034940510005752. 16087486

[6] Schlienger JL, Luca F, Vinzio S, Pradignac A [Obesity and cancer]. [Article in French]. Rev Med Interne. 2009; 30:776–82. 10.1016/j.revmed.2009.04.007. 19524333

[7] Travier N, Velthuis MJ, Steins Bisschop CN, van den Buijs B, Monninkhof EM, Backx F, Los M, Erdkamp F, Bloemendal HJ, Rodenhuis C, de Roos MA, Verhaar M, ten Bokkel Huinink D, et al Effects of an 18-week exercise programme started early during breast cancer treatment: A randomised controlled trial. BMC Med. 2015; 13:121. 10.1186/s12916-015-0362-z. 26050790 PMC4461906

[8] Dufresne S, Rébillard A, Muti P, Friedenreich CM, Brenner DR A review of physical activity and circulating miRNA expression: implications in cancer risk and progression. Cancer Epidemiol Biomarkers Prev. 2018; 27:11–24. 10.1158/1055-9965.EPI-16-0969. 29141851

[9] McTiernan A Mechanisms linking physical activity with cancer. Nat Rev Cancer. 2008; 8:205–11. 10.1038/nrc2325. 18235448

[10] Irwin ML, McTiernan A, Manson JE, Thomson CA, Sternfeld B, Stefanick ML, Wactawski-Wende J, Craft L, Lane D, Martin LW, Chlebowski R Physical activity and survival in postmenopausal women with breast cancer: results from the women’s health initiative. Cancer Prev Res (Phila). 2011; 4:522–29. 10.1158/1940-6207.CAPR-10-0295. 21464032 PMC3123895

[11] Izzotti A, Cartiglia C, Steele VE, De Flora S MicroRNAs as targets for dietary and pharmacological inhibitors of mutagenesis and carcinogenesis. Mutat Res. 2012; 751:287–303. 10.1016/j.mrrev.2012.05.004. 22683846 PMC4716614

[12] Zografos E, Zagouri F, Kalapanida D, Zakopoulou R, Kyriazoglou A, Apostolidou K, Gazouli M, Dimopoulos MA Prognostic role of microRNAs in breast cancer: a systematic review. Oncotarget. 2019; 10:7156–78. 10.18632/oncotarget.27327. 31903173 PMC6935258

[13] Altana V, Geretto M, Pulliero A MicroRNAs and physical activity. Microrna. 2015; 4:74–85. 10.2174/2211536604666150813152450. 26268469

[14] Nielsen S, Scheele C, Yfanti C, Akerström T, Nielsen AR, Pedersen BK, Laye MJ Muscle specific microRNAs are regulated by endurance exercise in human skeletal muscle. J Physiol. 2010; 588:4029–37. 10.1113/jphysiol.2010.189860. 20724368 PMC3000590

[15] Ni F, Gui Z, Guo Q, Hu Z, Wang X, Chen D, Wang S Downregulation of miR-362-5p inhibits proliferation, migration and invasion of human breast cancer MCF7 cells. Oncol Lett. 2016; 11:1155–60. 10.3892/ol.2015.3993. 26893711 PMC4734047

[16] Dethlefsen C, Hansen LS, Lillelund C, Andersen C, Gehl J, Christensen JF, Pedersen BK, Hojman P Exercise-induced catecholamines activate the hippo tumor suppressor pathway to reduce risks of breast cancer development. Cancer Res. 2017; 77:4894–904. 10.1158/0008-5472.CAN-16-3125. 28887324

[17] Dethlefsen C, Lillelund C, Midtgaard J, Andersen C, Pedersen BK, Christensen JF, Hojman P Exercise regulates breast cancer cell viability: systemic training adaptations versus acute exercise responses. Breast Cancer Res Treat. 2016; 159:469–79. 10.1007/s10549-016-3970-1. 27601139

[18] Chakravarthy MV, Joyner MJ, Booth FW An obligation for primary care physicians to prescribe physical activity to sedentary patients to reduce the risk of chronic health conditions. Mayo Clin Proc. 2002; 77:165–73. 10.4065/77.2.165. 11838650

[19] Neufer PD, Bamman MM, Muoio DM, Bouchard C, Cooper DM, Goodpaster BH, Booth FW, Kohrt WM, Gerszten RE, Mattson MP, Hepple RT, Kraus WE, Reid MB, et al Understanding the cellular and molecular mechanisms of physical activity-induced health benefits. Cell Metab. 2015; 22:4–11. 10.1016/j.cmet.2015.05.011. 26073496

[20] Koelwyn GJ, Quail DF, Zhang X, White RM, Jones LW Exercise-dependent regulation of the tumour microenvironment. Nat Rev Cancer. 2017; 17:620–32. 10.1038/nrc.2017.78. 28943640

[21] Stanford KI, Goodyear LJ Exercise and type 2 diabetes: molecular mechanisms regulating glucose uptake in skeletal muscle. Adv Physiol Educ. 2014; 38:308–14. 10.1152/advan.00080.2014. 25434013 PMC4315445

[22] Koh JH, Hancock CR, Han DH, Holloszy JO, Nair KS, Dasari S AMPK and PPARβ positive feedback loop regulates endurance exercise training-mediated GLUT4 expression in skeletal muscle. Am J Physiol Endocrinol Metab. 2019; 316:E931–E939. 10.1152/ajpendo.00460.2018. 30888859 PMC6580175

[23] Russell AP, Lamon S Exercise, skeletal muscle and circulating microRNAs. Prog Mol Biol Transl Sci. 2015; 135:471–96. 10.1016/bs.pmbts.2015.07.018. 26477927

[24] Pulliero A, Fazzi E, Cartiglia C, Orcesi S, Balottin U, Uggetti C, La Piana R, Olivieri I, Galli J, Izzotti A The aicardi-goutières syndrome. Molecular and clinical features of RNAse deficiency and microRNA overload. Mutat Res. 2011; 717:99–108. 10.1016/j.mrfmmm.2011.03.018. 21524657

[25] Izzotti A, Calin GA, Arrigo P, Steele VE, Croce CM, De Flora S Downregulation of microRNA expression in the lungs of rats exposed to cigarette smoke. FASEB J. 2009; 23:806–12. 10.1096/fj.08-121384. 18952709 PMC2653990

[26] Izzotti A, Pulliero A The effects of environmental chemical carcinogens on the microRNA machinery. Int J Hyg Environ Health. 2014; 217:601–27. 10.1016/j.ijheh.2014.01.001. 24560354

[27] Neubauer O, Reichhold S, Nics L, Hoelzl C, Valentini J, Stadlmayr B, Knasmüller S, Wagner KH Antioxidant responses to an acute ultra-endurance exercise: impact on DNA stability and indications for an increased need for nutritive antioxidants in the early recovery phase. Br J Nutr. 2010; 104:1129–38. 10.1017/S0007114510001856. 20637132

[28] Yan B, Zhu CD, Guo JT, Zhao LH, Zhao JL miR-206 regulates the growth of the teleost tilapia (Oreochromis niloticus) through the modulation of IGF-1 gene expression. J Exp Biol. 2013; 216:1265–69. 10.1242/jeb.079590. 23197102

[29] Min W, Wang B, Li J, Han J, Zhao Y, Su W, Dai Z, Wang X, Ma Q The expression and significance of five types of miRNAs in breast cancer. Med Sci Monit Basic Res. 2014; 20:97–104. 10.12659/MSMBR.891246. 25047098 PMC4117676

[30] Melov S, Tarnopolsky MA, Beckman K, Felkey K, Hubbard A Resistance exercise reverses aging in human skeletal muscle. PLoS One. 2007; 2:e465. 10.1371/journal.pone.0000465. 17520024 PMC1866181

[31] Joyce DP, Kerin MJ, Dwyer RM Exosome-encapsulated microRNAs as circulating biomarkers for breast cancer. Int J Cancer. 2016; 139:1443–48. 10.1002/ijc.30179. 27170104

[32] Zhou J, Tian Y, Li J, Lu B, Sun M, Zou Y, Kong R, Luo Y, Shi Y, Wang K, Ji G miR-206 is down-regulated in breast cancer and inhibits cell proliferation through the up-regulation of cyclinD2. Biochem Biophys Res Commun. 2013; 433:207–12. 10.1016/j.bbrc.2013.02.084. 23466356

[33] Rodríguez-González FG, Sieuwerts AM, Smid M, Look MP, Meijer-van Gelder ME, de Weerd V, Sleijfer S, Martens JW, Foekens JA MicroRNA-30c expression level is an independent predictor of clinical benefit of endocrine therapy in advanced estrogen receptor positive breast cancer. Breast Cancer Res Treat. 2011; 127:43–51. 10.1007/s10549-010-0940-x. 20490652

[34] Dobson JR, Taipaleenmäki H, Hu YJ, Hong D, van Wijnen AJ, Stein JL, Stein GS, Lian JB, Pratap J Hsa-mir-30c promotes the invasive phenotype of metastatic breast cancer cells by targeting NOV/CCN3. Cancer Cell Int. 2014; 14:73. 10.1186/s12935-014-0073-0. 25120384 PMC4129468

[35] De Santi M, Baldelli G, Lucertini F, Natalucci V, Brandi G, Barbieri E A dataset on the effect of exercise-conditioned human sera in three-dimensional breast cancer cell culture. Data Brief. 2019; 27:104704. 10.1016/j.dib.2019.104704. 31720346 PMC6838928

[36] Georgantas RW 3rd, Streicher K, Luo X, Greenlees L, Zhu W, Liu Z, Brohawn P, Morehouse C, Higgs BW, Richman L, Jallal B, Yao Y, Ranade K MicroRNA-206 induces G1 arrest in melanoma by inhibition of CDK4 and cyclin D. Pigment Cell Melanoma Res. 2014; 27:275–86. 10.1111/pcmr.12200. 24289491

[37] Li Y, Hong F, Yu Z Decreased expression of microRNA-206 in breast cancer and its association with disease characteristics and patient survival. J Int Med Res. 2013; 41:596–602. 10.1177/0300060513485856. 23696595

[38] Zhao N, Yu H, Yu H, Sun M, Zhang Y, Xu M, Gao W MiRNA-711-SP1-collagen-I pathway is involved in the anti-fibrotic effect of pioglitazone in myocardial infarction. Sci China Life Sci. 2013; 56:431–39. 10.1007/s11427-013-4477-1. 23633075

[39] Avellino R, Carrella S, Pirozzi M, Risolino M, Salierno FG, Franco P, Stoppelli P, Verde P, Banfi S, Conte I miR-204 targeting of Ankrd13A controls both mesenchymal neural crest and lens cell migration. PLoS One. 2013; 8:e61099. 10.1371/journal.pone.0061099. 23620728 PMC3631221

[40] Marchi S, Lupini L, Patergnani S, Rimessi A, Missiroli S, Bonora M, Bononi A, Corrà F, Giorgi C, De Marchi E, Poletti F, Gafà R, Lanza G, et al Downregulation of the mitochondrial calcium uniporter by cancer-related miR-25. Curr Biol. 2013; 23:58–63. 10.1016/j.cub.2012.11.026. 23246404 PMC3540261

[41] Jia W, Eneh JO, Ratnaparkhe S, Altman MK, Murph MM MicroRNA-30c-2* expressed in ovarian cancer cells suppresses growth factor-induced cellular proliferation and downregulates the oncogene BCL9. Mol Cancer Res. 2011; 9:1732–45. 10.1158/1541-7786.MCR-11-0245. 22024689

[42] Matsubara H, Takeuchi T, Nishikawa E, Yanagisawa K, Hayashita Y, Ebi H, Yamada H, Suzuki M, Nagino M, Nimura Y, Osada H, Takahashi T Apoptosis induction by antisense oligonucleotides against miR-17-5p and miR-20a in lung cancers overexpressing miR-17-92. Oncogene. 2007; 26:6099–105. 10.1038/sj.onc.1210425. 17384677

[43] Sacconi A, Biagioni F, Canu V, Mori F, Di Benedetto A, Lorenzon L, Ercolani C, Di Agostino S, Cambria AM, Germoni S, Grasso G, Blandino R, Panebianco V, et al miR-204 targets bcl-2 expression and enhances responsiveness of gastric cancer. Cell Death Dis. 2012; 3:e423. 10.1038/cddis.2012.160. 23152059 PMC3542596

[44] Abdelmohsen K, Kim MM, Srikantan S, Mercken EM, Brennan SE, Wilson GM, Cabo Rd, Gorospe M miR-519 suppresses tumor growth by reducing HuR levels. Cell Cycle. 2010; 9:1354–59. 10.4161/cc.9.7.11164. 20305372 PMC3057889

[45] Calzia D, Ottaggio L, Cora A, Chiappori G, Cuccarolo P, Cappelli E, Izzotti A, Tavella S, Degan P Characterization of C2C12 cells in simulated microgravity: possible use for myoblast regeneration. J Cell Physiol. 2020; 235:3508–18. 10.1002/jcp.29239. 31549411 PMC13482651

[46] Esquela-Kerscher A, Trang P, Wiggins JF, Patrawala L, Cheng A, Ford L, Weidhaas JB, Brown D, Bader AG, Slack FJ The let-7 microRNA reduces tumor growth in mouse models of lung cancer. Cell Cycle. 2008; 7:759–64. 10.4161/cc.7.6.5834. 18344688

[47] Ro YS, Shin SD, Song KJ, Hong KJ, Ahn KO Association of exercise and metabolic equivalent of task (MET) score with survival outcomes after out-of-hospital cardiac arrest of young and middle age. Resuscitation. 2017; 115:44–51. 10.1016/j.resuscitation.2017.03.041. 28389240

