# Supplementary material for: Anticancer effect of physical activity is mediated by modulation of extracellular microRNA in blood
Source: Oncotarget. 2020 Jun 2;11(22):2106–19. doi: 10.18632/oncotarget.27609 (PMC7275780; doi:10.18632/oncotarget.27609)
Supplement: Supplementary file 1 [file oncotarget-11-2106-s001.pdf]

Anticancer effect of physical activity is mediated by modulation of extracellular microRNA in blood

SUPPLEMENTARY MATERIALS

Supplementary Table 1: Relationships between miRNA expression intensity and clinical variables

MicroRNA	Glucose decrease (yes vs no)	Pre-heart rate (low vs high)	Borg Scale (low vs high)	Blood pressure (diastolic at 35 min) Increase (no vs yes)	Blood pressure (systolic at 35 min) increase (no vs yes)	Heart Rate (35 min) increase (no vs yes)
miR-25	↓		↓			
miR-30c	↓			↓		
miR-92	↓					↓
miR-133				↓	↓	
miR-204			↓	↓		
miR-206	↑			↓	↓	
miR-450				↓		
miR-492		↑	↓	↓	↓	
miR-513	↓					↓
miR-516	↓	↑	↓	↓		
miR-519	↓	↑	↓	↓		
miR-711	↓	↑	↓			
miR-765	↓					
miR-877		↑	↓	↓		
miR-1255	↓					↓
Total miRNA number	10	6	7	9	3	3

Subjects were categorized (yes vs no; low vs high) according to the 50° percentile of the clinical variable tested.
